# Hydroxytyrosol and Its Potential Uses on Intestinal and Gastrointestinal Disease

**DOI:** 10.3390/ijms24043111

**Published:** 2023-02-04

**Authors:** Alessia Arangia, Ylenia Marino, Daniela Impellizzeri, Ramona D’Amico, Salvatore Cuzzocrea, Rosanna Di Paola

**Affiliations:** 1 Department of Chemical, Biological, Pharmaceutical and Environmental Sciences, University of Messina, 98166 Messina, Italy; 2 Department of Veterinary Sciences, University of Messina, 98168 Messina, Italy

**Keywords:** hydroxytyrosol, intestinal diseases, gastrointestinal diseases, antioxidant proprieties, anti-inflammatory proprieties

## Abstract

In recent years, the phytoconstituents of foods in the Mediterranean diet (MD) have been the subject of several studies for their beneficial effects on human health. The traditional MD is described as a diet heavy in vegetable oils, fruits, nuts, and fish. The most studied element of MD is undoubtedly olive oil due precisely to its beneficial properties that make it an object of interest. Several studies have attributed these protective effects to hydroxytyrosol (HT), the main polyphenol contained in olive oil and leaves. HT has been shown to be able to modulate the oxidative and inflammatory process in numerous chronic disorders, including intestinal and gastrointestinal pathologies. To date, there is no paper that summarizes the role of HT in these disorders. This review provides an overview of the anti-inflammatory and antioxidant proprieties of HT against intestinal and gastrointestinal diseases.

## 1. Introduction

Over the last few years, the concept that food can act as medicine has been getting more and more acceptance by the scientific community. It is well known, in fact, that nutrition is capable of substantially modifying the risk profile of the population at both primary and secondary levels of prevention. Different types of diets have gained public attention, but the one that got the greatest interest is certainly the Mediterranean diet (MD). The MD was first described by Ancel Keys around the 1960s following the results of an epidemiological study [1]. In particular, Keys and colleagues demonstrated that olive-growing regions bordering the Mediterranean Sea had a reduced incidence of cardiovascular disease and cancer compared to other populations [1]. This observation has been confirmed by several subsequent studies, which provide strong evidence for the benefits of MD on cardiovascular health, including the reduction in the incidence of cardiovascular outcomes and risk factors, including obesity, hypertension, metabolic syndrome, and dyslipidemia [2,3,4,5]. Not only evidence from long-term observational studies but also clinical and preclinical studies demonstrated that the MD, closely linked to traditional areas of olive cultivation in the Mediterranean region, has been associated with low rates of chronic diseases, reductions in the incidence of some cancers, and neurodegenerative diseases; consequently, MD provides high adult life expectancy [2,6,7,8,9]. The traditional MD is described as a diet heavy in vegetable oils and low in saturated fat; it is commonly characterized by a high intake of fresh or minimally refined foods, such as cereals, vegetables, fruits, nuts, legumes, fish, olive oil, and red wine in moderation [9,10]. Individual foods and components of MD (e.g., extra-virgin olive oil and nuts) have numerous health-promoting properties [11,12,13,14,15,16], as they are rich in polyphenols, flavonoids, phenolic acids, and other bioactive compounds. Indeed, polyphenols are well-documented and can act as a free radical scavenger or metal chelator [17,18,19]. Thus, MD could trigger changes in signaling pathways and subsequent gene expression; in particular, it appears to act by modulating anti-inflammatory and antioxidant pathways [20,21,22]. Additionally, recent research has revealed that MD has antibacterial benefits in addition to lowering blood cholesterol levels and enhancing endothelial function [23,24]. 

It should be emphasized that MD is characterized by the regular intake of extra virgin olive oil (EVOO), whose significant beneficial effects are attributed to its phenolic constituents, fatty acid composition, and carotenoids such as lutein and beta-carotene [25]. One phenolic compound is hydroxytyrosol (HT), considered one of the most powerful antioxidant compounds among phenolic compounds from olive trees, followed by oleuropein, caffeic, and tyrosol (TYR) [25,26]. Due to its molecular structure (4-(2-hydroxyethyl)-1,2-benezediol), its regular consumption has several beneficial effects such as antioxidant, anti-inflammatory, antidiabetic, antimicrobial, anticancer, and as a protector of skin and eyes, etc. [27,28,29]. In this regard, the nutraceutical and food industries employ HT as a nutritional supplement due also to its high bioavailability and lack of toxicity [30,31]. Growing knowledge of the beneficial effect of HT has led researchers to investigate it as a therapeutic agent for chronic disorders, especially inflammatory bowel diseases (IBDs) [32]. IBDs are a group of immune-based disorders characterized by prolonged inflammation and severe intestinal dysbiosis resulting in severe damage to the gastrointestinal tract [32,33]. To date there are no papers that summarize the effects of HT in these pathologies; therefore, the objective of this review is to provide an overview of the anti-inflammatory and antioxidant effects of HT against intestinal and gastrointestinal diseases.

## 2. Hydroxytyrosol

### 2.1. Source of Hydroxytyrosol

**Figure 1:**
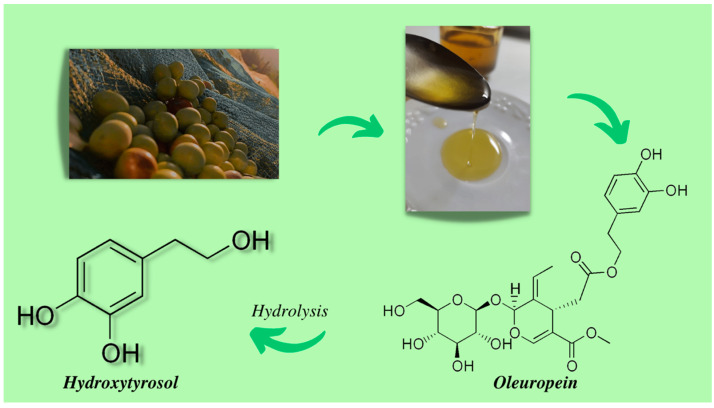
The chemical structure of hydroxytyrosol, a derivative of the hydrolysis of oleuropein contained in olive oil.

HT is a polyphenol with potent antioxidant capacity due to its ortho-dihydroxy conformation in the aromatic ring (Figure 1). It is a member of the catechol class and is a primary alcohol. HT and TYR are obtained through the hydrolysis of oleuropein, which is the main constituent that gives olives their bitter taste. This process of oleuropein hydrolysis occurs during olive ripening, storage, and processing [34]. In fact, HT is the most abundant metabolite found in extra virgin olive oil, olives (*Olea europea* L.), and wine. What makes the study of HT interesting is its ability to induce the synthesis of antioxidant enzymes, as well as its ability to scavenge reactive oxygen species (ROS) [35]. Furthermore, Napolitano et al. have shown that HT is able to donate hydrogen ions, and this gives it a better ability to chelate iron compared to TYR [36].

### 2.2. Extraction and Purification of Hydroxytyrosol

A high concentration of HT is found in olive leaves; however, this is lost as a residue during oil production. To reduce industrial waste, the residue is used to obtain natural products used in the food, cosmetics, and pharmaceutical industries [26]. HT is obtained from vegetation water (i.e., waste water deriving from the processing of olive oil) or leaves through three steps. The first step consists of obtaining liquid rich in phenols which are extracted and purified. In the second step, an extract and mixture of HT with 3,4-dihydroxyphenlglycol are obtained, and HT acetate is produced. In the end, these components are made highly pure. In industrial processes, different sources of extraction are used; for example, olive waters are produced during processing by centrifuge/separation of the oil. The leaves, on the other hand, are dehydrated and undergo a process of hydroalcoholic extraction and hydrolysis. From this hydroalcoholic solution, oleuropein is obtained, which in turn is purified from its hydrolysis by HT [37]. Papageorgiou and colleagues described the purification processes of HT in detail [38]. Here, we summarize that HT can be recovered through solid–liquid extraction, acid hydrolysis, or liquid–liquid extraction with ethyl acetate [38].

### 2.3. Absorption, Distribution, Metabolism, and Excretion (ADME) of Hydroxytyrosol

Phenolic compounds are absorbed in a dose-dependent manner and undergo intestinal-hepatic metabolism. Specifically, HT is absorbed and excreted differently depending on how it is taken (aqueous or oily) [10]. The average absorption of HT is about 66% and occurs in the small intestine and colon by passive transport through the intestinal epithelium [25]. The uptake of HT present in wine is different. Indeed, Perez-Mana et al. have highlighted how the interaction between HT and ethanol may result in a change in dopamine metabolism that induces the production of HT instead of producing DOPAC (3,4-dihydroxyl phenylacetic acid) [10,39]. In any case, absorption is rapid, and a maximum plasma concentration is reached 5–10 min after intake, followed by a rapid decline. After absorption, HT binds to circulating lipoproteins, acting as an antioxidant and cardiovascular protector [40]. Furthermore, HT is easily distributed in various organs such as the kidney, liver, skeletal muscle, lungs, and heart. Not only, but HT is also able to cross the blood-brain barrier, thereby reaching the brain and interacting with dopaminergic pathways, as observed in a study by D’Angelo through radioactively labeling HT by intravenous administration in rats [41]. Regarding metabolism, HT suffers the first step inside enterocytes and subsequently in the liver [10]. Both steps are important because HT undergoes several modifications and transformations, presumably endowing it with its beneficial properties. Briefly, HT metabolism can be summarized according to three reactions: oxidation, methylation, and methylation-oxidation, as described by Ramírez-Tortose et al. [34]. Finally, HT and its metabolites are excreted through the urine within 6 h in humans and within 4 h in rats. Excretion could vary depending on the administration to humans or animals or even according to quantity or vehicle [10]. Obviously, all this affects the dosage of HT used for different pharmacological and nutraceutical purposes [34].

### 2.4. Toxicity and Dose Establishment

Due to its chemical properties, HT is used as a dietary supplement in the nutraceutical and food industries. In fact, it is an excellent scavenger and protects against lipid peroxidation. HT is the main component of olive oil studied to date and is responsible for numerous beneficial effects. Regarding its toxicity and safety profile, there are several studies done on olive mill wastewater and olive oil [10]. According to several studies based on the different doses of HT, no toxic effect of HT was observed both in acute and subchronic toxicity [27,34]. Therefore, based on the observations made, it was concluded that HT does not exhibit genotoxic and mutagenic effects, as stated in in vitro models by Aunon-Calles et al. [10]. In fact, it has been seen that HT has no toxic effects but has beneficial effects such as anti-apoptotic, antiproliferative, antioxidant, and anti-inflammatory activity and acts as a cardiovascular protector. So toxicity is not relevant, but the European Food Safety Authority suggests taking 5 mg of HT in order for it to perform its activity as an anti-inflammatory, antioxidant, and low-density lipoprotein (LDL) oxidation inhibiter [34].

### 2.5. Proprieties of Hydroxytyrosol

**Figure 2:**
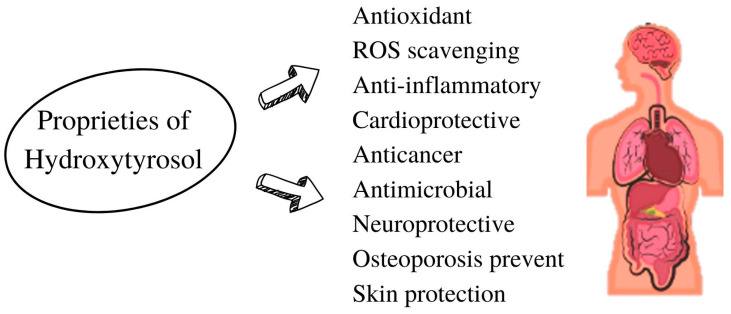
Principal proprieties of hydroxytyrosol.

HT has numerous beneficial effects, as described earlier, is a nonmutagenic compound, is not genotoxic, and has a high safety profile. The main effects of HT are described below (Figure 2).

#### 2.5.1. Antioxidant Proprieties and ROS Scavenging

HT exhibits a protective effect on the organism as it induces the production of antioxidant enzymes through modulation of the nuclear factor erythroid 2-related factor 2 (Nrf2) pathway. This involves the translocation of Nrf2 into the nucleus resulting in the expression of cytoprotective genes such as heme oxygenase (HO-1) [10,42]. It also induces the synthesis of proteins that lead to DNA repair or phase II detoxifying enzymes [25]. Indeed, HT is able to increase the antioxidant network, such as superoxide dismutase (SOD), glutathione peroxidase (GPx), and catalase (CAT), and keep reduced glutathione (GSH) levels constant [43]. This allows ROS to be eliminated in the cellular environment [10]. Furthermore, in an in vitro study by Rietjens et al., it has been shown that HT stimulates the activation of signaling pathways that recognize the presence of ROS [44], and HT can protect against LDL oxidation even at low concentrations.

#### 2.5.2. Anti-Inflammatory Proprieties

Inflammation is a form of protection against damaged cells and pathogens. Normally the inflammatory response involves the nuclear factor kappa-light-chain-enhancer pathway of activated B cells (NF-κB). HT may act as an anti-inflammatory agent by inhibiting nuclear translocation of NF-κB, thereby inhibiting the expression of genes involved in inflammation. In particular, HT blocks arachidonic acid (AA), cyclo-oxygenase, and lipoxygenase (COX and LOX), inhibits pro-inflammatory cytokines, such as TNF-α and IL-1β, and other enzymes involved in inflammation [26,45]. In addition, HT is able to suppress inducible nitric oxide synthase/nitric oxide. In a study by Scoditti et al. conducted on activated human monocytes, HT at appropriate concentrations suppresses the activation and expression of the enzymes MMP-9 and COX-2 [46]. Additionally, the lowered lymphocyte activity inhibited by the anti-inflammatory effects of HT could result in the development of novel ways for the treatment of inflammatory disorders [26].

#### 2.5.3. Anticancer Proprieties

HT has antiproliferative and hence apoptotic effects via chelating metal ions and scavenging free radicals [10,27]. In addition, studies carried out in vitro on HL60 cell lines (leukemia cell lines) revealed that HT behaved as a pro-apoptotic by causing the release of cytochrome c from the mitochondrial intermembrane gap and activating caspase 8 [47,48]. In vitro studies demonstrated that HT could inhibit ERK1/2 to reduce cell growth in MCF-7 (Michigan Cancer Foundation-7) breast cancer cells [27]. Chemento et al. showed that HT induces apoptosis of ER-negative SL-BR-3 breast cancer by acting as an antagonist for the G-protein-coupled estrogen receptor (GPER), leading to apoptosis [49]. Rosignoli and colleagues demonstrated that HT plays a different role on different cancer cell lines of MCF-7 (breast carcinoma), LNCap, and PC3 (prostate carcinoma), SW480, and HCT116 (colon carcinoma), in that in the culture medium, it leads to the accumulation of hydrogen peroxide, acting as a prooxidant-anti-tumor [50]. In addition, HT has been seen to lead to the inhibition of signaling pathways such as Akt, STAT3, and EGFR in pancreatic, prostate, and thyroid cancer cell lines [50,51].

#### 2.5.4. Antimicrobial Proprieties

In the past, microbial infections were cured by olive leaf extracts and olive oil. Properly in the leaves, there is a high concentration of HT. In fact, it has potent antioxidant activity against some microorganisms such as *Escherichia coli*, *Candida albicans*, *Clostridium perfringens*, *Streptococcus mutans,* and *Salmonella enterica* [27]. Furthermore, Bisignano et al. have highlighted that HT acts as an antibacterial, but it also has an antiparasitic effect [10]. The mode by which HT acts is to reduce ROS production, which is induced by microbial biofilms. There are contradictions regarding the effects of HT against bacteria and parasites, so further studies are needed.

#### 2.5.5. Other Proprieties of Hydroxytyrosol

Finally, several studies have identified other protective effects of HT important for human health. It was demonstrated that HT limits the development of atherosclerotic plaque, protects against LDL oxidation, and reduces the risk of cardiovascular disease [10]. The cardioprotective effect carried out by HT leads to the inhibition of aging-related proteins in cardiac cells [52]. Furthermore, HT is also neuroprotective, as it can cross the BBB and diffuses freely into central neurons. Here, it appears to inhibit monoamine oxidase (MAO) in patients with Parkinson’s disease, protecting against neurodegeneration [6,53,54]. Finally, HT protects against UV radiation, thus having a dermoprotective effect. Skin is exposed daily to ultraviolet radiation (UVR), increasing the formation of free radicals. Zwane et al. showed that HT and its metabolites act as radical scavengers for skin cells, and in addition, HT significantly reduces DNA breakdown caused by ultraviolet B [10]. Therefore, HT may exert different roles in chronic diseases.

## 3. Role of Hydroxytyrosol in IBDs

IBD is a global healthcare problem with a sustained increasing incidence, affecting millions of people worldwide, all ages and genders [55,56]. It includes two major forms, Ulcerative Colitis, and Crohn’s Disease, which are distinct chronic inflammatory disorders of the gastrointestinal tract with differences in the pathology and clinical characteristics [57,58]. Ulcerative Colitis is typified by mucosal inflammation and is limited to the colon. In contrast, Crohn’s Disease can cause transmural inflammation and affect any part of the gastrointestinal tract (most commonly, the terminal ileum or the perianal region) in a non-continuous type [59,60]. It is unclear what causes IBD, but it is thought to be caused by a combination of factors, including genetic, environmental, and especially dietary variables [61,62,63]. The association between nutrition and IBD has been extensively studied. Dietary fat has been found to play a role in the IBD pathogenesis [64,65,66]; on the contrary, patients with an MD lifestyle, with a high intake of dietary fibers, such as fruits, vegetables, and especially EVOO, are associated with reduced risk for both Ulcerative Colitis and Crohn’s Disease [67,68,69,70]. This is probably because the underlying pathogenetic mechanism of IBD involves the alteration of the redox balance [71,72]; the increased oxidative stress leads to the propagation of inflammation and exacerbation of mucosal damage in the gastrointestinal tract [73,74,75]. Below, we report the in vitro and in vivo findings, along with clinical results, supporting the protective role of HT against IBD due to its antioxidant and anti-inflammatory activity.

### 3.1. Hydroxytyrosol in Ulcerative Colitis

Ulcerative colitis is one of the most common inflammatory bowel diseases [20], characterized by abdominal and/or rectal pain, diarrhea, bloody stools, weight loss, fever, and even rectal prolapse in severe cases. Ulcerative colitis can also progress to colon cancer [76,77]. It has been widely demonstrated that a dysfunction of the immune system underlies the development of colitis [78,79]. The gastrointestinal tract is the target of various harmful bacteria and substances, and immune cells usually mount an immune-inflammatory response that neutralizes them. However, in ulcerative colitis, this response does not occur [80,81]; this leads to damage to the intestinal mucosa and submucosa (in severe disease, the muscularis is also involved), with consequent loss of integrity of the intestinal barrier [82,83]. The immune response is usually mediated by macrophages and dendritic cells [84]. These cells have TLR receptors on the surface of the membrane, responsible both [85] for the differentiation of T-cells, which in turn stimulate the synthesis of proinflammatory cytokines, but they also induce the activation of the transcription factor NF-κB, further exacerbating the inflammatory process [84,86,87,88]. The key factors involved in the inflammatory cascade during ulcerative colitis are cytokines, such as tumor necrosis factor-α (TNF-α), interferon-γ (IFN-γ), interleukin (IL) -2, IL-6, IL-8, which are also linked to chemokines and adhesion molecules [89,90]. HT has been shown to have protective and immunomodulatory effects against intestinal inflammation in several in vivo and in vitro experimental models [25,91,92]. In particular, HT appears to act by modulating the NF-κB signaling pathway, the consequent release of pro-inflammatory cytokines, and the expression of molecules downstream of the inflammatory cascade, such as COX-2 and iNOS [32,91,93,94,95,96,97]. Another study demonstrated that HT could exert anti-inflammatory effects in DSS-induced ulcerative colitis by inhibiting NLRP3 inflammasome activation and modulating gut microbiota in vivo [98]. Furthermore, ulcerative colitis is associated with a disequilibrium between the oxidant and antioxidant system, giving rise to the overproduction of ROS, which contributes to worsening the complications of the pathology [86,99,100]. In recent years, both in vivo and in vitro studies have been conducted confirming the antioxidant activity of HT also against ulcerative colitis. These effects are probably due to the fact that HT is able to promote Nrf2 nuclear localization [101,102]. The nrf2 signaling pathway is important for maintaining redox homeostasis and defending against ROS by inducing antioxidant enzyme synthesis [103,104]. Indeed, HT can also increase antioxidant enzyme expressions, such as SOD, CAT, and GPx, and at the same time diminish lipid peroxidation marker malondialdehyde (MDA) [93,101,105]. In addition, in vitro studies confirmed the antioxidant effect of HT using human intestinal Caco-2 cells [91,96,106]. Lastly, in a study by Elmaksoud et al., HT has been shown to modulate the apoptotic process by downregulation of the apoptotic gene Bax and upregulation of the anti-apoptotic gene Bcl2 [93]. Randomized crossover clinical trials have also been performed investigating the effect of HT, which is the main component of EVOO, in UC patients [94,107,108]. The promising results from the studies reproduce many of the effects seen in animal and in vitro studies, such as anti-inflammatory effects, gastrointestinal symptom reduction, and positive microbiota changes [32]. However, we must be cautious in translating these findings into a real-life clinical setting.

### 3.2. Hydroxytyrosol in Crohn’s Disease

As in ulcerative colitis, also in the development of Crohn’s disease, there is an incorrect regulation of the immune system and the response mediated by TLR receptors [109]. The NOD receptors of innate immunity are often found next to the TLR receptors, which under normal conditions, induce the release of NF-κB and cytokines to eliminate agents harmful to the intestinal mucosa [110,111]. In Crohn’s disease, however, these receptors induce a persistent chronic inflammatory condition that leads to excessive release of inflammatory cytokines such as IL-1β, IL-2, IL-3, IL-22, and TFN-α [109]. Furthermore, it is thought that there is also an alteration of the adaptive immune response and B-cells, but this mechanism is not yet fully understood. Certainly, it has been seen and confirmed that the inability of T-cells to undergo apoptosis results in T-cell accumulation and mucosal damage [112]. In the inflammatory environment, HT inhibits the release of TNF-α and proinflammatory mediators, and, in addition, HT also acts on T-cells, as demonstrated by Kawaguchi et al. [113,114]. Additionally, the commonly observed effects to elucidate the molecular basis for HT’s anti-inflammatory activity in Crohn’s disease were inhibition of p38/MAPK and NF-κB signaling pathways and reduction of iNOS expression and NO release [115,116,117]. These results were also confirmed in a study by Vezza et al. [95] that investigated the ex vivo organ cultures of mucosal explants from CD patients, showing a reduction of the proinflammatory mediators’ production. Furthermore, it should be emphasized that an alteration of the redox balance also occurs in Crohn’s disease with excessive production of ROS [73]. In this regard, HT has been defined as a strong antioxidant and free radical scavenger in several studies investigating Crohn’s disease [118,119]. To date, the clinical trials in the literature are preliminary data but are certainly promising at this early stage [40,120].

## 4. Role of HT in Other Gastrointestinal Diseases

### 4.1. Hydroxytyrosol in Gastric Ulcer

Gastric ulcer is a disease that affects many people around the world; in most cases, the underlying cause is infection sustained by Helicobacter pylori, long-term use of anti-inflammatory drugs, and other factors such as smoking, alcohol drinking, and dietary [121,122]. The causes mentioned above cause an imbalance in the normal mucosal barrier, altering the production of gastric mucus that acts as a protective layer for the underlying epithelium, from gastric juice and pepsin [123,124,125]. As we have already described above, HT also has an antimicrobial action. The reason why HT has been shown through in vitro studies to have beneficial effects on gastric ulcers as it acts against the proliferation of Helicobacter pylori. Not only that, HT was able to reduce the size of the ulcer as well [32,126,127]. Studies further confirmed that HT inhibits NF-κB transcription, thereby reducing the release of inflammatory cytokines, including TNF-α [128,128]. Furthermore, in an in vivo study by Arismendi Sosa et al., HT demonstrated not only antioxidant and antimicrobial effects but also improved gastric mucosal damage [127]. A number of clinical trials are being developed to demonstrate the anti-inflammatory and antimicrobial effects of HT on gastric ulcers [129].

### 4.2. Hydroxytyrosol in Colorectal Cancer

Colon-rectal cancer is a disease that affects many people around the world. It is a pathology characterized by a certain aggressiveness due to the ability of the tumor to proliferate and invade the surrounding tissues. Mutations and genetic modifications affecting DNA repair systems underlying the molecular mechanism of colorectal cancer development have been identified [130,131]. In recent years, higher consumption of olive oil, rich in polyphenols such as hydroxytyrosol, has been associated with low incidence and prevalence of cancer, including colorectal cancer [132,133,134,135]. The beneficial action of HT has been evaluated through in vitro studies on epithelial and carcinogenic cell lines: it has been demonstrated that HT was able to act on genes involved in apoptosis, such as Bax and Bcl-2, Caspase-3, and p53 [47,136]. Additionally, recent studies have evidenced that HT inhibits the cell proliferation of colon cancer cells by activating the estrogen receptor-β (ERβ) [51]. In fact, it has been proven that cancerous progression in human colon mucosa is characterized by a reduction in ERβ expression [133]. Finally, Hormozi et al. [47] demonstrated that HT upregulates antioxidative activity in the colorectal cancer cell line through a significant increase of antioxidant enzymes. However, in vivo studies on the therapeutic effects of HT on colorectal cancer in animal models can give better conclusions. Indeed clinical studies have shown how HT affects the mechanisms involved in the development of this type of cancer [137].

### 4.3. Hydroxytyrosol in Gastroesophageal Reflux Disease and Eosinophilic Esophagitis

Gastroesophageal reflux is a chronic and relapsing disorder occurring when the stomach contents reflux into the esophagus, causing troublesome symptoms that have a negative impact on the quality of life of patients. The cause that leads to the development of this disorder is the dysfunction of the esophageal sphincter, which does not close properly [138,139]. In addition, Barrett’s esophagus (development of columnar metaplasia in place of the normal squamous epithelium), esophageal strictures, and esophageal adenocarcinoma are conditions linked to gastrointestinal reflux [140,141]. Another pathology affecting the esophagus is eosinophilic esophagitis, which is often confused with gastroesophageal reflux. This is a chronic immune-mediated inflammatory disease of the esophagus, still poorly understood, that causes dysphagia, food impaction of the esophagus, and esophageal strictures [142]. Studies have shown that damage to the mucosa of the esophagus leads to a chronic inflammatory condition, with the release of proinflammatory cytokines and often also the formation of fibrotic tissue in both conditions [143,144]. To date, it has been shown that a balanced diet with a high intake of polyphenols can prevent the development of these disorders [145,146,147]. Last year, a double-blinded randomized-controlled trial assessed the efficacy of extracts from *O. europaea* leaves (rich in bioactive compounds such as HT and tyrosol) in gastrointestinal discomfort, showing a decrease in frequency and intensity of the main symptoms of this disorder, such as abdominal swelling, heartburn, and belching [148]. The protective effects may be due to the active compounds present in the extract, which exert a protective action on gastric mucosa together with the protection from oxidative damage and associated complications and through the modulation of the inflammatory response [148,149,150]. The results of the study by Malfa and colleagues [148] were positive, but further supporting studies are needed.

## 5. Conclusions

In conclusion, we can state that the main phytoconstituent of the olive tree, HT has excellent potential for use on human health. The various studies carried out so far have shown that HT has excellent bioavailability; in fact, it is easily absorbed and metabolized by the human body. Furthermore, it has been demonstrated, both in vitro and in vivo, that HT is not toxic, genotoxic, or mutagenic. In this review, we have highlighted the potential role of HT in modulating the molecular mechanisms underlying the development of IBDs and gastrointestinal diseases. This is made possible as HT has shown to have a strong antioxidant and free radical scavenger action, as it increases the activity of antioxidant enzymes and restores the oxidative balance. Among its main proprieties, HT also has an anti-inflammatory effect, mainly due to the inhibition of the NF-κB pathway and the release of inflammatory cytokines. Furthermore, it is important to underline that HT has an immunomodulatory and antimicrobial, but also pro-apoptotic and anti-proliferative action, which is why it appears to act against colorectal cancer. This could explain the beneficial effects that HT shows towards these multifactorial pathologies. Therefore, due to its beneficial properties, good safety profile, and numerous protective effects, HT should be further investigated in order to be used in clinical conditions.

## Data Availability

Not applicable.
